# Changes in bone macro- and microstructure in diabetic obese mice revealed by high resolution microfocus X-ray computed tomography

**DOI:** 10.1038/srep35517

**Published:** 2016-10-19

**Authors:** G. Kerckhofs, M. Durand, R. Vangoitsenhoven, C. Marin, B. Van der Schueren, G. Carmeliet, F. P. Luyten, L. Geris, K. Vandamme

**Affiliations:** 1 Skeletal Biology and Engineering Research Center, Department of Development and Regeneration, KU Leuven, 3000 Leuven, Belgium; 2 Prometheus - Division of Skeletal Tissue Engineering Leuven, KU Leuven, 3000 Leuven, Belgium; 3 UMR CNRS 7052, Biomécanique et Biomatériaux Ostéo-Articulaires, Faculté de Médecine Lariboisière, 75000 Paris, France; 4 Institut de Recherche Biomédicale des Armées (IRBA), Département Soutien Médico-Chirurgical des Forces (SMCF), 91220 Brétigny-sur-Orge, France; 5 Clinical and Experimental Endocrinology, Department of Clinical and Experimental Medicine, KU Leuven, 3000 Leuven, Belgium; 6 Division of Biomechanics and Engineering Design, KU Leuven, 3001 Heverlee, Belgium; 7 Biomechanics Research Unit, University of Liège, 4000 Liège, Belgium; 8 Biomaterials – BIOMAT, Department of Oral Health Sciences, KU Leuven, 3000 Leuven, Belgium

## Abstract

High resolution microfocus X-ray computed tomography (HR-microCT) was employed to characterize the structural alterations of the cortical and trabecular bone in a mouse model of obesity-driven type 2 diabetes (T2DM). C57Bl/6J mice were randomly assigned for 14 weeks to either a control diet-fed (CTRL) or a high fat diet (HFD)-fed group developing obesity, hyperglycaemia and insulin resistance. The HFD group showed an increased trabecular thickness and a decreased trabecular number compared to CTRL animals. Midshaft tibia intracortical porosity was assessed at two spatial image resolutions. At 2 μm scale, no change was observed in the intracortical structure. At 1 μm scale, a decrease in the cortical vascular porosity of the HFD bone was evidenced. The study of a group of 8 week old animals corresponding to animals at the start of the diet challenge revealed that the decreased vascular porosity was T2DM-dependant and not related to the ageing process. Our results offer an unprecedented ultra-characterization of the T2DM compromised skeletal micro-architecture and highlight an unrevealed T2DM-related decrease in the cortical vascular porosity, potentially affecting the bone health and fragility. Additionally, it provides some insights into the technical challenge facing the assessment of the rodent bone structure using HR-microCT imaging.

Diabetes Mellitus (DM) affects 56.3 million individuals in Europe and about 387 million worldwide (http://www.idf.org/). The current pandemic of the most common type of diabetes, type 2 diabetes (T2DM), largely results from a lifestyle with low physical activity and a high caloric diet leading to obesity. Obesity-induced T2DM is characterized by a progressive development of insulin resistance in liver and peripheral tissues accompanied by a defective insulin secretion from pancreatic beta cells leading to overt hyperglycaemia. Chronic hyperglycaemia results in microvascular complications (diabetic nephropathy, neuropathy, and retinopathy) as well as macrovascular morbidity (coronary artery disease, peripheral arterial disease, and stroke) and ultimately increased mortality^1^. Substantial progress in diabetes monitoring and treatment has significantly increased the life expectancy of patients. As patients live longer, other comorbidities related to the diabetic condition have emerged, including a compromised skeletal health^2^. Indeed, obese patients with T2DM experienced a 40 to 70% increased fracture risk despite a paradoxal normal to relatively high bone mineral density (BMD) compared to control subjects^3^,^4^,^5^,^6^. These fractures are particularly problematic because T2DM patients also exhibit longer and impaired fracture healing and poorer outcomes after fracture^7^. The mechanisms underlying the poor skeletal health in T2DM patients is currently not well understood, but is likely to be multifactorial and to include deficits in both bone material properties and bone macro- and microstructure. Indeed, one of the potential accounting mechanism is the deterioration of the bone matrix due to the accumulation of advanced glycation end products (AGEs)^8^. Recently, Poundarik *et al*. showed a marked reduction in toughness and indentation measures in diabetic mice exhibiting increased AGEs levels^9^.

Regarding the bone macro- and microstructure, no consensus has been reached on the influence of T2DM on the trabecular bone architecture. Moreover, although most of the fractures in obesity induced-T2DM occur at sites that are rich in cortical bone^10^, the impact of T2DM on the cortical compartment is poorly understood. In both human and animal studies, cortical bone thickness has been reported to increase^11^,^12^, decrease^13^,^14^,^15^, or remain unchanged^16^,^17^,^18^. Also changes in intracortical porosity are highly controversial. For instance, Burghardt^17^ and others^14^,^19^ reported increased cortical porosity in postmenopausal women with T2DM. However, another study indicated that cortical porosity is increased only in postmenopausal diabetics with a prior fragility fracture, and that diabetics without a prior fracture had similar cortical bone porosity as non-diabetic controls^20^. The cortical bone porosity, which includes the vascular canals and the osteocyte lacunar system, is an important contributor of the skeletal health. On one hand, it is a determinant of bone strength and stiffness and might predict prevalent fractures independently of BMD^21^,^22^. On the other hand, the vascular supply to bone, in relation with the blood flow and nutrient supply, plays an important role in the maintenance and the healing of the tissue^23^. Therefore, given the incomplete understanding of the contributing factors to the poor skeletal health in T2DM, it is crucial to characterize the full 3D macro- and microstructure of the diabetic bone. As animal models are valuable tools for the investigation of diabetic complications, the present study aimed at exploring the influence of T2DM on the bone structural properties in the high fat diet (HFD) mouse model of obesity-driven T2DM. This well-described animal model closely parallels the common course of the human disease by firstly develop obesity so as to finally develop diabetes. 3D-investigation of the T2DM bone structural alterations was performed by using a desktop high resolution microfocus X-ray computed tomography (HR-microCT) approach. Not only this technique allows assessing the trabecular and cortical bone macrostructure, but it also provides quantification of the microstructure by isolating the vascular canal porosity independently from the lacunar porosity in the cortex at high resolution in a time and cost saving way.

Changes in the serum levels of bone turnover markers as well as detection of the cell death were also determined. Altogether, data gleaned from the present study provide a better characterization of the T2DM compromised skeletal macro- and micro-architecture and, more specifically, highlight unrevealed T2DM-related changes in the cortical vascular porosity.

## Results

### Physiological parameters

As seen in Table 1, HFD mice had an obese phenotype evidenced by a 37.2% increased body weight compared to CTRL littermates (p < 0.001). HFD animals also showed significant fasting hyperglycaemia (+89.7%, p < 0.001), along with a massive rise in HOMA-IR index (8.3-fold augmentation, p < 0.001) when compared to CTRL animals. The body weight in the CTRL group was significantly higher than that in the YNG group (p < 0.001). While the fasting blood glucose level was significantly lower (p = 0.041), the HOMA-IR was not statistically different (p = 0.058).

### Bone macrostructure and bone remodelling

#### Trabecular macrostructure

**Figure 1:**
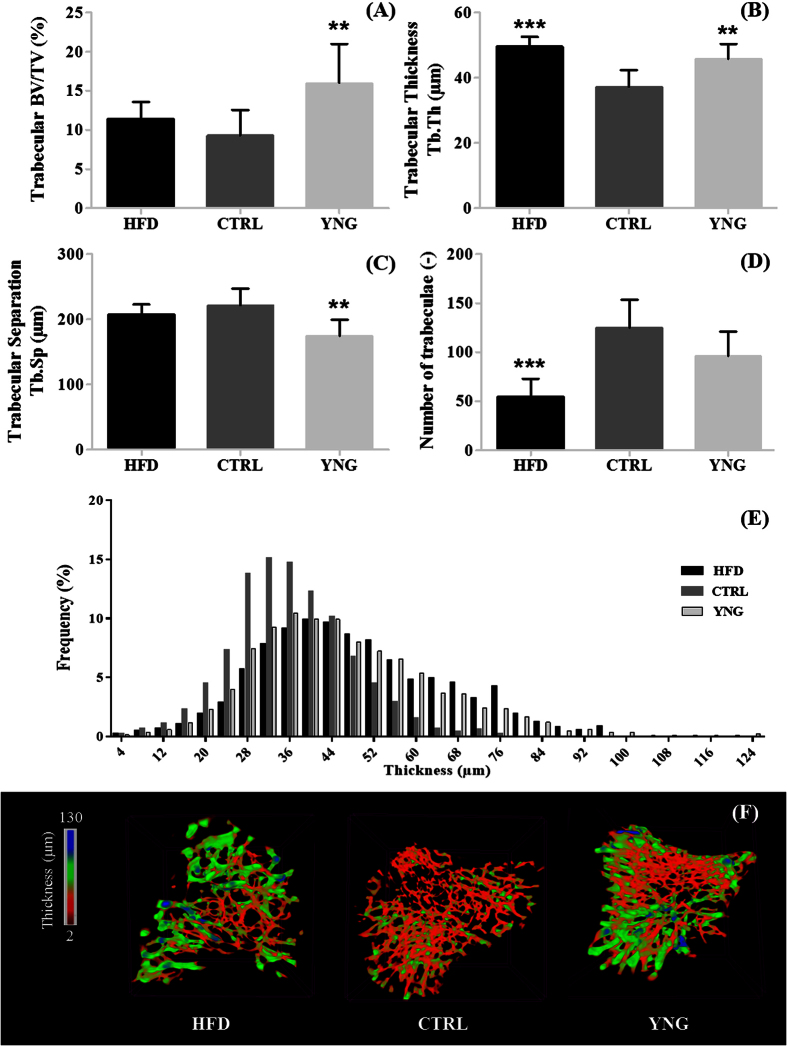
HR-microCT-based analysis of the (**A**) trabecular bone volume fraction, (**B**) trabecular thickness, (**C**) trabecular separation and (**D**) number of trabeculae for the HFD, CTRL and YNG groups. (**E**) The distribution plot of the trabecular thickness for the HFD, CTRL and YNG groups. (**F**) Typical color-coded 3D renderings of the trabecular architecture representing the thickness of the trabeculae. n = 7–8/group.

Having checked the diabetic profile of the HDF animals, we then investigated the changes in trabecular architecture related to obesity-driven T2DM, and alternatively associated them with the ageing process (Fig. 1). Examination of the proximal tibia revealed that mice fed the HFD had significantly less (Fig. 1D) but thicker (Fig. 1B) trabeculae (−56.2%; p < 0.001 and +33.0%; p < 0.001, respectively) compared to CTRL littermates although the spacing between the trabeculae (Fig. 1C) had not changed significantly (p = 0.247). This resulted in an unchanged bone volume fraction (p = 0.165 – Fig. 1A). When compared to YNG mice, a lower trabecular BV/TV was evidenced in CTRL mice (9.28 ± 1.16% and 16.48 ± 1.96% for CTRL and YNG respectively, p = 0.007 – Fig. 1A) due to a significantly reduced trabecular thickness (p = 0.005 – Fig. 1B) and increased trabecular separation (p = 0.004 – Fig. 1C), but an unchanged trabeculae number (p = 0.059 – Fig. 1D). The decrease in trabecular thickness for the CTRL group when compared to HFD or to YNG mice is clearly illustrated on the thickness distribution plot (Fig. 1E) and on the color-coded 3D renderings of the thickness of the trabecular bone (Fig. 1F).

#### Cortical macrostructure

**Figure 2:**
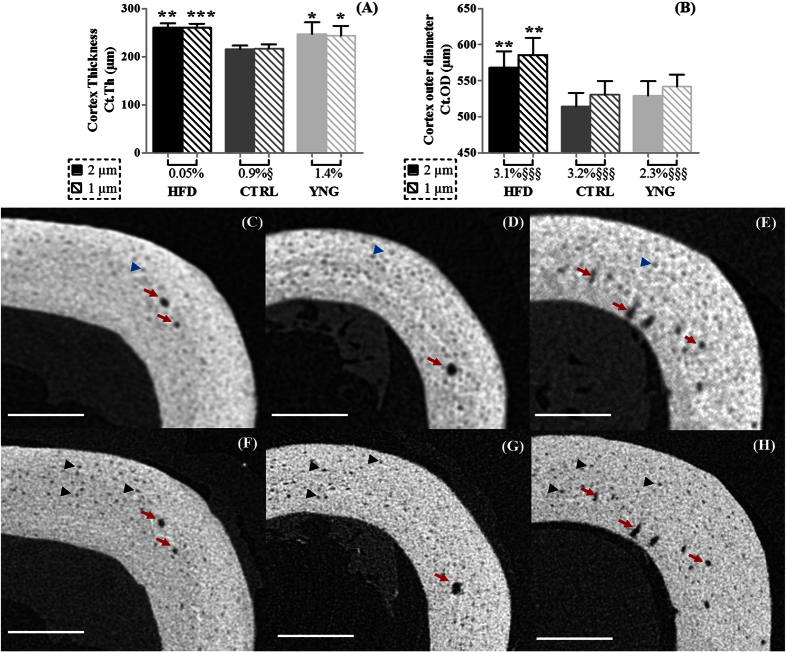
HR-microCT-based analysis of the (**A**) cortex thickness and (**B**) cortex outer diameter. Statistical comparison has been made between the different animal groups (HFD and YNG versus CTRL), indicated with an asterisk for both scanning resolutions, and between the 2 μm and 1 μm voxel size scans per animal group, indicated with an §. The percentage under the bar graph per animal group represents the relative difference between the 2 μm and 1 μm voxel size scans. Typical cross-sectional HR-microCT images of the cortex of a (**C,F**) HFD, (**D,G**) CTRL and (**E,H**) YNG animal scanned at (**C–E**) 2 μm voxel size and (**F–H**) 1 μm voxel size. Red arrows indicate vascular canals, black arrows indicate osteocyte lacunae that can be segmented and quantified and blue arrows indicate osteocyte lacunae that cannot be segmented from the cortical bone. Scale bars = 200 μm, n = 7–8/group.

Regarding the cortical macrostructure (Fig. 2), both the 2 μm and the 1 μm voxel size scans showed that the Ct.Th was higher in HFD animals than in CTRL control animals (+ 21.1% for 2 μm scans, p < 0.001 and +20.0% for 1 μm scans, p < 0.001 – Fig. 2A). In an obesity-driven T2DM-free environment, i.e. in normal ageing (CTRL group vs. YNG group), the Ct.Th decreased significantly by 13.6% for the 2 μm scans (p = 0.012) and by 10.9% for the 1 μm scans (p = 0.013). There was no significant difference between the 2 μm and 1 μm voxel size results for the Ct.Th in the different groups, except for the CTRL group. In this group, the Ct.Th at 2 μm voxel size was significantly underestimated (p = 0.031) by 0.9% when compared to 1 μm voxel size scans. When the cortex outer diameter was concerned, both the 2 μm and 1 μm voxel size scans showed a significant increase for the HFD group compared to the CTRL littermates (10.5%, p = 0.001 and 10.4%, p = 0.001 respectively – Fig. 2B), although there were significant differences (p < 0.001) between the 2 μm and 1 μm voxel size results for all the groups. The increase in the Ct.OD was not noticed in a normal ageing environment.

The differences between the 2 μm and 1 μm voxel size scans are a result of the partial volume effect, which is smaller at higher resolution leading to a sharper bone surface (Fig. 2C–H). Although there were significant differences between the 1 μm and 2 μm voxel size scans, the higher accuracy of the images did not reveal additional differences between the animal groups.

### Bone resorption and bone formation

**Figure 3:**
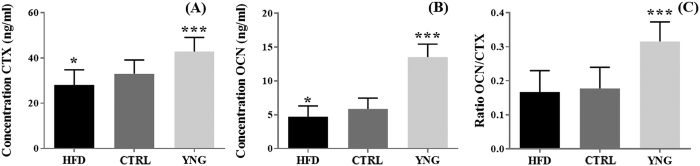
Elisa-based measurements of the (**A**) CTX and (**B**) OCN expression in murine blood serum, and (**C**) the ration of (**B**) over (**A**). n = 6–8/group.

Given the macrostructural alterations, we next investigated the rate of bone turnover in our mice by measuring serum C-terminal telopeptide of type 1 collagen (CTX - Fig. 3A) and serum osteocalcin (OCN - Fig. 3B), biomarkers for bone resorption and formation respectively. When comparing YNG with CTRL animals, more bone resorption was noticed for the YNG group (29.7%, p < 0.001), but also more bone formation (131.0%, p < 0.001), hence indicating more bone remodelling (Fig. 3C; 78.0%, p < 0.001). The latter correlates well with the higher trabecular BV/TV (Pearson’s correlation coefficient = 0.94 – Fig. 1A). In HFD animals, there is less bone resorption (14.9%, p = 0.014), but also less bone formation (19.9%, p = 0.014) compared to the age-matched littermates. This might be linked with the increased trabecular thickness (Fig. 1B), and the decreased trabecular number (Fig. 1D) for the HDF group. They have only slightly less bone remodelling compared to the CTRL group (5.8%, p = 0.63 – Fig. 3C), which again correlates well with the trabecular BV/TV being not significantly different for both groups (Fig. 1A).

### Bone vascularity

#### Microstructural characterization

**Figure 4:**
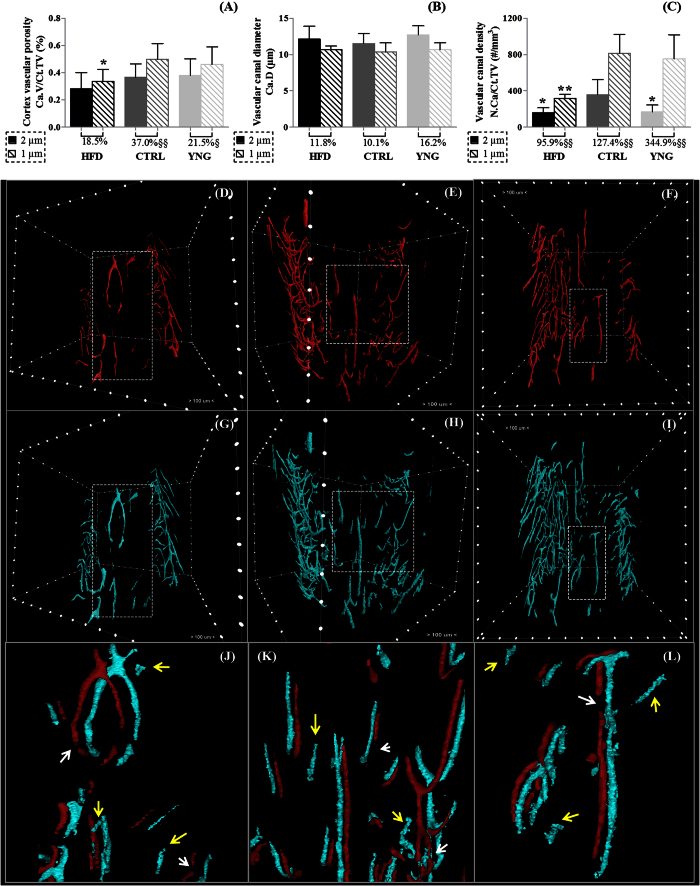
HR-microCT-based analysis of the (**A**) cortex vascular porosity, (**B**) vascular canal diameter and (**C**) vascular canal density for the HFD, CTRL and YNG groups. Statistical comparison has been made between the different animal groups (HFD and YNG versus CTRL, indicated with an asterisk) for both scanning resolutions, and between the 2 μm and 1 μm voxel size scans (indicated with an §) per animal group. The percentage under the bar graph per animal group represents the relative difference between the 2 μm and 1 μm voxel size scans. Representative 3D renderings of the vascular canals of a (**D,G**) HFD, (**E,H**) CTRL and (**F,I**) YNG animal scanned at (**D–F**) 2 μm voxel size and (**G–I**) 1 μm voxel size. As indicated, distance between the marks on the bounding box is 100 μm. Zoom of the dotted area in the 2 μm and 1 μm voxel size images of a (**J**) HFD, (**K**) CTRL and (**L**) YNG animal, where the red canals represent the 2 μm voxel size scan and the blue canals the 1 μm voxel size scan. Missing connections in the 2 μm voxel size scans are indicated with white arrows and canals only present in the 1 μm voxel size scans are indicated with yellow arrows. n = 7–8/group.

When evaluating the vascular canal system in the cortex at 2 μm voxel size (Fig. 4), there was no significant difference in the average vascular canal porosity (p = 0.225 – Fig. 4A) neither in the vascular canal diameter (p = 0.535 – Fig. 4B) in HFD animals compared to the CTRL group, despite a significant lower vascular canal density (−55.0% decrease in HFD animals, p = 0.034 – Fig. 4C). In normal ageing, only the density of the vascular canals was found significantly increased in CTRL animals (p = 0.021 – Fig. 4C).

**Figure 5:**
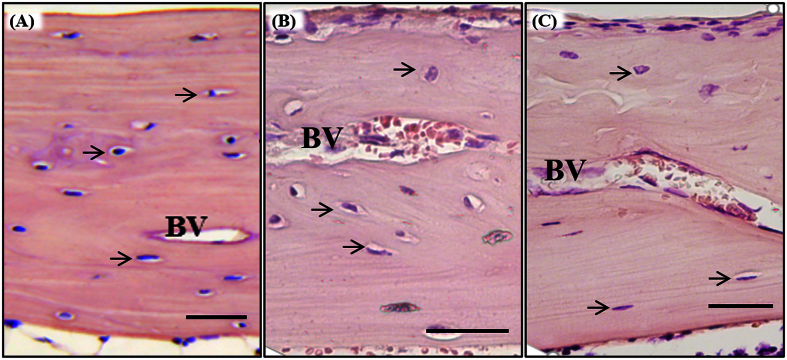
Representative H&E-stained histological sections of the cortex (longitudinal sections) of a (**A**) HFD, (**B**) CTRL and (**C**) YNG mouse. ‘BV’ indicates blood vessels where the erythrocytes are clearly visible, while the black arrows point out osteocyte lacunae. Scale bar = 100 μm.

In contrast, the datasets acquired at 1 μm voxel size revealed a significant drop (−32.7%; p = 0.019) in vascular canal porosity in HFD mice when compared to CTRL controls. The HFD group also exhibited a lower vascular canal density (61.2% lower than CTRL group, p = 0.002), whereas the diameter of the canals remained unchanged (p = 0.572). Vascular canal parameters in CTRL animals were not significantly different from those in the YNG group, suggesting that the compromised vascular cortical porosity in HFD mice was likely to result from the obesity-driven T2DM environment rather than from ageing. Histological examination of H&E stained sections corroborated HR-microCT data (Fig. 5).

Paired t-tests highlighted the important effect of the spatial image resolution on the quantification of the vascular porosity. The largest influence was seen for the vascular canal density. When compared to the 1 μm voxel size scans, the vascular canal density at 2 μm voxel size was underestimated by 95.9% for the HFD group (p = 0.005), by 127.4% for the CTRL group (p = 0.004) and by 344.9% (p = 0.001) for the YNG group. Inspections of the datasets revealed that the 2 μm voxel size images (Fig. 4D–F) produced a less continuous delineation of the vascular canals (Fig. 4J–L – white arrows) than the 1 μm voxel size images (Fig. 4G–I) and lacked the thinner canals (Fig. 4J–L – yellow arrows). Vascular canals also appeared thinner at 2 μm voxel size than at 1 μm voxel size, resulting in a reduction by 11.8%, 10.1% and 16.2% in HDF (p = 0.104), CTRL (p = 0.001) and YNG (p = 0.33) animals respectively in the vascular canal diameter. Consequently, the cortical vascular porosity at 2 μm voxel size was largely underestimated in the three groups (−18.3% for HFD, p = 0.352, −36.9% for CTRL, p = 0.001 and −21.5% for YNG, p = 0.033) when compared to the 1 μm voxel size.

### Bone osteocyte lacunar system

#### Microstructural analysis

**Figure 6:**
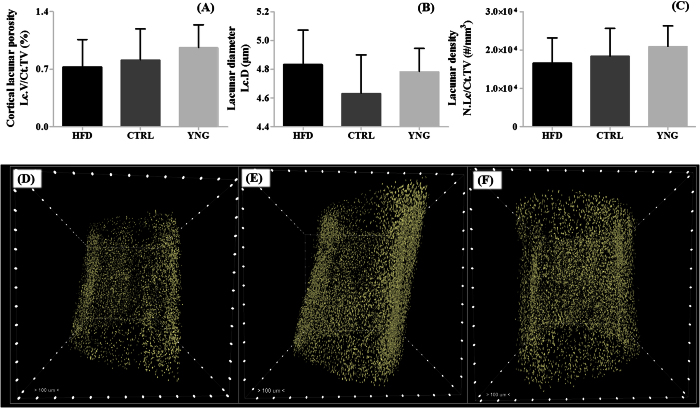
HR-microCT-based analysis of the (**A**) cortex lacunar porosity, (**B**) lacunar diameter and (**C**) lacunar density for the HFD, CTRL and YNG groups. Typical 3D renderings of the lacunar system of a (**D**) HFD, (**E**) CTRL and (**F**) YNG. As indicated, distance between the marks on the bounding box is 100 μm. n = 7–8/group.

Analysis of the lacunar system at 1 μm voxel size using a despeckling volume of 280 μm^3^, including the lacunar porosity (Fig. 6A), density (Fig. 6B) and diameter (Fig. 6C), did not reveal significant differences neither in the obesity-driven T2DM environment (HFD *vs.* CTRL animals) nor in normal ageing (CTRL *vs.* YNG animals). This was also confirmed in the 3D renderings of the images (Fig. 6D–F). A decreasing trend in lacunar porosity and density could however be noticed due to ageing, and also when comparing the HFD group with the CTRL controls. Additionally, although not statistically significant, the lacunar diameter was on average lower for the CTRL group compared to both the YNG and the HFD group.

#### Viability of the osteocytes - TUNEL staining

**Figure 7:**
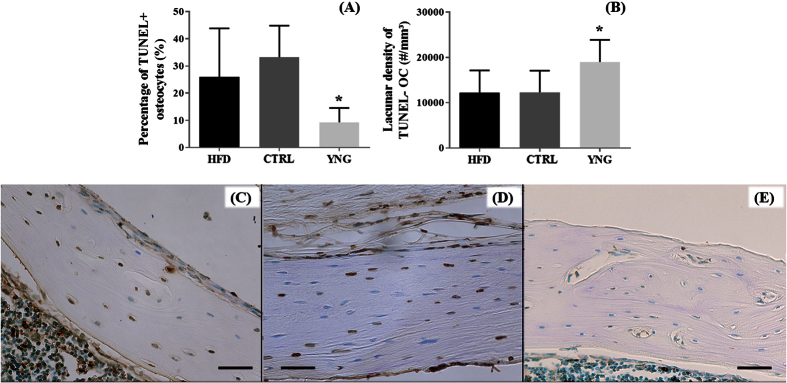
TUNEL-based measurements of the (**A**) percentage of TUNEL+ osteocytes and (**B**) the lacunar density of TUNEL− osteocytes (using HR-microCT data) for HFD, CTRL and YNG animals. n = 3–4/group. Typical TUNEL-stained cross-sections of the cortex of a (**C**) HFD, (**D**) CTRL and (**E**) YNG mouse. Brown osteocytes are TUNEL+ and are apoptotic. Blue osteocytes are TUNEL−. Scale bars = 50 μm.

Following the characterization of the osteocyte lacunar system, we then investigated the integrity of the cells within the lacunae by assessing osteocyte viability. Prevalence of apoptotic TUNEL positive osteocytes within the cortex is shown on Fig. 7C–E. Quantitative analysis of these images indicated a significantly lower percentage of TUNEL-positive osteocytes for the YNG group compared to the CTRL group (9.26 ± 5.29% and 33.22 ± 11.64% respectively; p = 0.031 – Fig. 7A), while no significant differences were found between the CTRL and the HFD group (25.97 ± 17.87%, p = 0.59). When combining the percentage of TUNEL-negative osteocytes with the HR-microCT-based lacunar density (Fig. 7B), a significant difference appeared when comparing the CTRL with the YNG group (12285.41 ± 4806.74 osteocytes/mm^3^ and 18990.67 ± 4888.98 osteocytes/mm^3^ respectively, p = 0.031). CTRL and HFD animals (12227.53 ± 4916.64 osteocytes/mm^3^) showed to have a similar density of TUNEL-negative osteocytes (p = 0.99).

## Discussion

T2DM is a complex and multifactorial disease largely resulting from a Western style diet and an excessive weight. T2DM does not spare the skeletal system and is associated with bone fragility and poor bone healing^24^,^25^. We hypothesized that a contributing factor to these impairments would be the alteration in vasculature and architecture of the bone. Therefore, we assessed the macro- and microstructure of T2DM bone in the HFD–fed C57BL/6J mouse model in a detailed and 3D manner using HR-microCT, and substantiated these results using histomorphometry and immunoassays.

Our findings revealed T2DM related changes in the structural pattern of the trabecular and the cortical compartments. The use of a group of young animals allowed us to assess the mere age-induced effects on bone homeostasis in the current experiments. Indeed, in comparison with YNG animals, CTRL mice displayed an osteopenic trabecular bone phenotype characterized by a decreased trabecular bone volume fraction and thickness and an increased separation, along with a decreased bone resorption and formation (as indicated by the CTX and OCN expression). This trabecular bone loss bears a remarkable resemblance to age-related changes in humans^26^. HFD mice had a similar trabecular bone mass to that of the CTRL animals, which is in contrast to several earlier studies^13^,^15^,^27^, but supports the recent works from Doucette *et al*. and Lecka Czernik *et al*.^18^,^28^. Although HFD animals showed a similar ratio of OCN/CTX, they did have a significantly decreased bone resorption and formation compared to the CTRL controls, as also has been indicated by Starup-Linde *et al*. 29 The decreased Tb.N in the HFD mice confirms all the previous studies in the field regardless the duration of the HFD or the bone piece^15^,^16^,^18^,^30^. Changes in trabecular thickness in response to the HFD in mouse tibia are conflicting in the literature^16^,^28^,^30^.

Because changes in cortical porosity were found to be “ageing-independent” in our study, we can assume that the decrease in vascular porosity was related to the HFD phenotype of obesity-driven T2DM. More research needs to be done to investigate whether the impaired vascular porosity, which may contribute to the poor bone healing in the T2DM environment^31^, is driven by the direct action of HFD on angiogenesis, resulting in an alteration of the bone architecture, or whether HFD primarily affects bone cells, subsequently leading to defective vasculature.

In contrast to our work, HR-pQCT-based studies in human suggested that T2DM in postmenopausal women is associated with abnormal high cortical porosity^14^,^17^,^19^. Interestingly however, although cortical porosity is associated with increased bone turnover^32^,^33^, a number of studies paradoxically showed reduced bone turnover in patients with T2DM^34^,^35^, similar to our own findings (i.e. CTX and OCN measurements). In 2013, new elements were provided by Patsch *et al*. by reporting that changes in porosity are dependent of the history of fractures^20^. They found evidence that cortical porosity was only strongly related to a prior history of fracture in those with diabetes and was not a characteristic of diabetes in general. Indeed, cortical porosity tented to be lower although not statistically different in diabetics without fragility fractures than healthy controls. In light of these observations, our results, that seemed inconsistent with literature at first, provided new insight into the assessment of the human diabetic bone.

A limitation of the present study is the HFD model itself, which does not allow separating the effects of hyperglycaemia per se, insulin resistance, excess body weight and ageing on the skeletal response. Mechanical testing has indicated a significant decrease in the bending stiffness of HFD bones compared to the CTRL group (Supplementary data – Supplementary Figure S1). However, in order to determine what the mechanisms are behind the decreased mechanical properties, and whether this decrease is the result of the altered macro- and micro-architecture, or a change in the bone composition due to the decreased cortical vascularization, and hence lower nutrient supply, or a combination of both, additional experiments need to be performed. The use of computational modelling, additional mechanical testing including micro- or nano-indentation, bone compositional analysis or the study of a non-transgenic mouse model, obese but free of metabolic disorder, would be helpful in dissecting the underlying mechanisms of the T2DM bone mechanical alterations.

Our study also showed the important influence of the spatial image resolution on the quantification of the vascular system in the cortex. Although scanning at a 2 μm voxel size has been suggested to be appropriate for measuring the vascular porosity^36^, we have shown that this resolution was not sufficient to pick up the smaller vascular canals and resulted in a significant underestimation of the vascular canal density and porosity of the cortex. As a consequence, the difference in the vascular porosity between the HFD group and the CTRL controls was only picked up at 1 μm voxel size. This resolution has indeed been shown to be relevant when assessing vascular and lacunar porosities in rats^36^ and in mice^37^. Compared to these studies, however, where the scanning times went up to about 5 hours per sample, in our study the scanning time was only 20 minutes per sample, making routine analysis possible in a time and cost saving way^38^. The short scanning time in our study also prevented drying of the sample during scanning, which not only could have an influence on the image quality (i.e. movement artefacts), but also could affect subsequent immunohistological analysis of the samples, adding to the potential value of the use of HR-microCT. It is important to highlight that the HR-microCT measurements, without the use of a contrast agent, only allow visualizing and quantifying the vascular cortical porosity and not the blood vessels as such. As a consequence, it is not possible to discern permanent blood vessels from remodelling units, or to exclude that in some canals no blood vessels are present.

One of the major concerns in the study of the T2DM-related complications is the relevance of the model in regards to the human condition. Since obesity is the major environmental factor predisposing to T2DM, we selected a rodent model recognized to closely parallel the common course of the human disease by firstly develop obesity so as to finally develop diabetes^39^. However, the development of overt diabetes is controversial in this animal model and the vulnerability of C57BL/6J individuals to the HFD is variable^40^,^41^. Therefore, we controlled the main manifested pictures of T2DM in our animals by showing a rise in fasting glucose level, insulin resistance and body weight in mice fed the HFD. Given the mechanosensory role of osteocytes and their newly recognized endocrine function^42^, we further explored the osteocyte and its lacunar system, expecting to see changes in the HFD animals. Despite a lack of statistical evidence, our results regarding the lacunar density, scanned at 1 μm voxel size, followed a slight downward trend but similar to that seen in the work of Lai and co-workers in a spontaneous type 1 diabetic (T1DM) mouse model^43^. The authors suggested that the decrease is possibly due to the impaired osteoblast differentiation commonly associated with diabetes. However, apart from the considerable standard deviation on the results due to differences between animals within one group, it is possible that the spatial resolution (1 μm voxel size) used in this study for the quantitative assessment of the osteocyte lacunar porosity was not sensitive enough to pinpoint differences among the experimental groups. The use of synchrotron X-ray computed tomography^38^, allowing also an increased contrast resolution, could be a solution to validate the results from this study and to determine whether the osteocyte lacunar porosity is modified due to T2DM or not.

As expected, apoptotic osteocytes were found higher in the cortical bone of the CTRL group compared to the YNG group. Recently, it has been shown that treatment of osteocyte like MLO-Y4 cells with high glucose concentrations and AGEs induces their apoptosis^44^. Besides, previous *in vivo* studies indicated that excessive loading increases the prevalence of osteocyte apoptosis^45^,^46^. Hence, our TUNEL assay showing a high rate of dead osteocytes in the HFD animals was not surprising, albeit we would have expected a higher prevalence of apoptotic osteocytes in HFD than CTRL animals. Osteocyte apoptosis controls the activation of the intracortical bone remodelling by triggering osteoclast-mediated bone resorption^47^. It would be interesting to test whether or not a longer high fat diet challenge would be associated with a higher prevalence of TUNEL+ osteocytes and related bone resorption.

In conclusion, our HR-microCT analysis indicates that obesity-driven T2DM induced by a 14 week-HFD affects both macro- and microstructure of bone in C57BL/6J mice, and this is corroborated by histomorphometry and immunoassays. Specifically, it is associated with a thickening and widening of the cortical bone and thickening of the trabeculae along with a decrease in their number, and a reduced bone resorption and formation. Moreover, we show that obesity-driven T2DM compromises the intracortical vascular porosity. This unprecedented ultra-characterization can provide novel insight into the T2DM-related compromised skeletal macro- and micro-architecture, and illustrates in addition the potential added value of HR-microCT imaging when assessing the macro- and microstructure of the rodent bone.

## Materials and Methods

### Animal care and diets

All animal procedures were performed with approval of the Medical and Animal Ethics Committee of the KU Leuven, in accordance with the ARRIVE guidelines and the ILAR Guide to the Care and Use of Experimental Animals. Inbred male C57BL/6 mice were housed with free access to water in a temperature- and light-controlled vivarium housing room on a 12-hour light/dark cycle. Three groups were assessed in this study. At 8 weeks of age, mice were randomly assigned to the high fat diet group (HFD group, n = 7) or the age-matched control group (CTRL group, n = 8) and were fed either a high fat diet (60% energy as fat, Ref No D12492, Open Source Diets, Research Diets Inc., New Brunswick, NJ, USA) or normal chow (11% energy as fat, Ref No S8189-S085, Ssniif, Germany) respectively. Mice were fed ad libitum for a period of 14 weeks, at which time point they were sacrificed. To evaluate the effect of ageing, a third group of lean young mice fed normal chow was sacrificed at 8 weeks of age (YNG group, n = 8). All animals were euthanized through intracardiac puncture under isoflurane anaesthesia and tissues were collected for analysis.

### Physiological assessment of T2DM

Obesity-driven T2DM was assessed at the time of sacrifice by measuring the body weight, the fasting glycaemia and the fasting insulin level. Mice were fasted for 6 hours prior to euthanasia. Fasting blood glucose levels were determined from tail vein blood samples using an Accu-Chek Aviva glucose meter (Roche Diagnostics, Vilvoorde, Belgium). Quantitative determination of fasting plasma insulin was performed by immunoassay (Mercodia Mouse Insulin Elisa, Uppsala, Sweden). Insulin resistance is defined as a state in which a normal or elevated insulin level produces an inadequate biological response. As a hallmark of T2DM, insulin resistance was assessed in our animals using the homeostasis model assessment (HOMA-IR) originally described by Hunter *et al*.^48^. HOMA-IR index scores were calculated as HOMA-IR = (FBG x FPI)/405, where FBG denotes fasting blood glucose (mg/dL) and FPI is fasting plasma insulin (mU/mL). The factor 405 accounts for the measurement unit.

### High resolution microfocus X-ray computed tomography (HR-microCT) – acquisition

**Figure 8:**
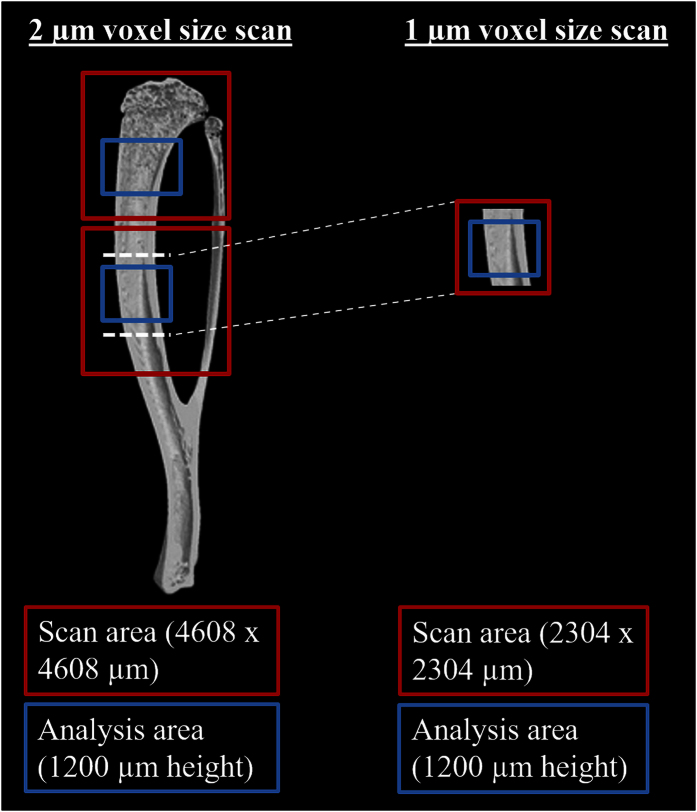
Schematic representation of the HR-microCT acquisition strategy: 2 μm voxel size scan versus 1 μm voxel size scan.

After harvesting, the tibias were fixed in a freshly prepared 4% paraformaldehyde solution for 1 day and transferred to phosphate-buffered saline (PBS) for storage. Then, they were scanned at two different spatial resolutions (Fig. 8) using HR-microCT (Phoenix NanoTom S, GE Measurement and Control Solutions).

First, the proximal tibiae were examined at 2 μm isotropic voxel size to assess the trabecular and cortical macrostructure. The source, equipped with a tungsten target, was operated at 70 kV and 120 μA. An aluminium filter of 0.3 mm was applied to reduce beam hardening. Because the ‘fast mode’ settings (i.e. exposure time 500 ms, frame averaging 1 and skip 0) were used, the scanning time was only 20 minutes per sample.

In order to be able to separate the intracortical lacunar-canalicular porosity surrounding osteocytes from the vascular porosity, the tibiae were then scanned at 1 μm voxel size (Fig. 8). The limited field of view (2.3 mm × 2.3 mm) at 1 μm isotropic voxel size did not allow scanning the entire tibia because of its curvature. Thus, tibias were cross-sectioned at mid-diaphysis. The source was set at 70 kV and 60 μA, and an aluminium filter of 0.3 mm was used to reduce beam hardening. Application of the ‘fast mode’ settings resulted again in a scanning time of 20 minutes per sample.

### High resolution microfocus X-ray computed tomography (HR-microCT) – image analysis

Images were analysed using CTAn (Bruker MicroCT, Kontich, Belgium). For the assessment of the trabecular architecture, we selected 600 images (1.2 mm height) starting at 800 μm below the growth plate level. On this dataset, a region of interest (ROI) was drawn manually incorporating the trabecular structure, but not including the cortical bone. Using automatic Otsu segmentation, the images were binarised, and noise was removed by a closing operation (round kernel, radius 1, 3D space) and a double despeckling step (removal of black and white speckles respectively, 3D space, less than 200 voxels). Using 3D analysis, the trabecular volume in the total ROI volume (BV/TV), trabecular number (Tb.N), thickness (Tb.Th) and separation (Tb.Sp.) were calculated. The trabecular thickness distribution was visualized in 3D using CTVox (Bruker MicroCT, Kontich, Belgium).

Analysis of the cortical macrostructure and porosity at mid-shaft tibia (vascular porosity in the 2 μm voxel size scans, and vascular and osteocyte lacunar porosity in the 1 μm voxel size scans) was performed on 1.2 mm high datasets (600 images for the 2 μm voxel size scans, and 1200 images for the 1 μm voxel size scans). First, both 2 μm and 1 μm scans were oriented in the same plane, using DataViewer (Bruker MicroCT, Kontich, Belgium). Then, we selected the same cortical midshaft region in order to be able to compare the 2 μm voxel size scans with the 1 μm voxel size scans. The ROI fitting the cortical bone was drawn automatically using an in-house developed protocol. Using manual global segmentation, the images were binarised for the pores, and noise was removed by a despeckling step, i.e. removal of white speckles in 3D space less than 280 μm^3^ (35 voxels for the 2 μm voxel size scans and 280 voxels for the 1 μm voxel size scans). To evaluate the sensitivity of the despeckling step on the cortical lacunar porosity, lacunar diameter and number of lacunae, and to determine the most optimal despeckling value for our study, we ranged the white speckle volume from 220 μm^3^ up to 340 μm^3^ (based on literature) in steps of 30 μm^3^, and also included more extreme values (150 μm^3^, 450 μm^3^ and 550 μm^3^). Based on these results, we selected a threshold of 280 μm^3^ as lower limit despeckling volume (Supplementary data - Supplementary Figure S2). All the remaining pores were assigned as vascular plus lacunar pores, and the cortical porosity was determined. After a following despeckling step to remove white speckles smaller than 4000 μm^3^, only the vascular pores remained, and the vascular canal density (number per mm^3^- N.Ca/Ct.TV, #/mm^3^), diameter (Ca.D, μm) and porosity (Ca.V/Ct.TV, %) were calculated. Finally, the dataset was reloaded, white speckles smaller than 280 μm^3^ and larger than 4000 μm^3^ were removed by despeckling, and the lacunar density (N.Lc/Ct.TV, #/mm^3^), diameter (Lc.D, μm) and porosity (Lc.V/Ct.TV, %) was determined. For the different steps, the binarised images of the pores were saved, and the vascular and lacunar (only for the 1 μm voxel size scans) porosity was visualized in 3D using CTVox. To determine the cortical thickness (Ct.Th, μm), the thickness of the ROI was calculated both for the 2 μm and 1 μm voxel size scans, as this fits perfectly around the cortex and does not include the cortical porosity. When then filling up the marrow cavity, the cortical outer diameter (Ct.OD, μm) was determined on the 2D sections based on the plate model where Ct.OD = 2/(surface/volume).

### Histology and histomorphometry

After HR-microCT imaging, decalcified samples were embedded in paraffin and sectioned (5 μm thick). Standard Haematoxylin and Eosin (H&E) staining was used for the visual inspection of the cortical vascular and lacunar porosity. Terminal deoxynucleotidyl transferase dUTP nick end labeling (TUNEL) staining was performed using the ApopTag^®^ peroxidise *In Situ* Apoptosis Detection Kit S7100 (Millipore, Guyancourt, France). In the counting of the TUNEL-positive and negative osteocytes, only osteocytes in the mid-diaphysis of tibias which were recognized based on their morphology were counted.

### Immunoassays

#### CTX and OCN ELISA

Serum from 6 animals per group was collected after the blood was allowed to clot for 2 h at room temperature. Serum C-terminal telopeptide of type 1 collagen (CTX) and serum osteocalcin (OCN) were measured using ELISA kits (i.e. RatLapsTM (CTX-I) EIA from Immunodiagnosticsystem and mouse Osteocalcin ELISA kit from Immunotopics, Inc. respectively).

### Statistical analysis

Data for all parameters are expressed as group means ± standard deviation (SD). Sample size calculation (n = 7–8 animals/group) was carried out using the resource equation method^49^. Analysis of normality of the data was performed using the Shapiro-Wilk test and the presence of outliers was tested using the Dixon test. Equality of variances was assumed when the F-test (normally distributed) or Levene’s test (not normally distributed) revealed values of p > 0.05. HFD group and YNG group were both independently compared to CTRL animals using a two-tailed unpaired t-test (normally distributed) or a Kruskal-Wallis test (not normally distributed) with equal or unequal variance depending on the F-test or Levene’s test respectively. Since the HFD model needs a combination of time (ageing) and diet to generate T2DM, we cannot assume that these factors are truly independent. For this reason, we could not perform a one-way ANOVA and could not compare the HFD with the YNG group. To compare the 2 μm voxel size scans with the 1 μm voxel size scans, a paired t-test was used. All statistical analysis was performed using Sigma Plot (Systat Software, Inc., San Jose, CA, USA). Significance is indicated as follows: *p < 0.05, **p < 0.01, and ***p < 0.001 when compared to the CTRL group; ^§^p < 0.05, ^§§^p < 0.01, and ^§§§^p < 0.001 when comparing the 2 μm voxel size scans with the 1 μm voxel size scans.

## Additional Information

**How to cite this article**: Kerckhofs, G. *et al*. Changes in bone macro- and microstructure in diabetic obese mice revealed by high resolution microfocus X-ray computed tomography. *Sci. Rep.* **6**, 35517; doi: 10.1038/srep35517 (2016).

## Supplementary Material

Supplementary Information

## Figures and Tables

**Table 1:** Body weight and metabolic parameters (mean ± SD) of HFD, CTRL and YNG animals.

	HFD	CTRL	YNG
Body weight (g)	42.80 ± 2.14***	31.19 ± 1.39	22.74 ± 1.24***
Glycaemia (mg/dL)	307.50 ± 53.26***	162.13 ± 24.35	197.71 ± 36.08*
HOMA-IR	42.75 ± 10.78***	5.14 ± 2.97	9.61 ± 4.59

n = 7-8/group.
