# Supplementary material for: Changes in bone macro- and microstructure in diabetic obese mice revealed by high resolution microfocus X-ray computed tomography
Source: Sci Rep. 2016 Oct 19;6:35517. doi: 10.1038/srep35517 (PMC5069481; doi:10.1038/srep35517)
Supplement: Supplementary Information [file srep35517-s1.pdf]

Changes in bone macro- and microstructure in diabetic obese mice revealed by high resolution microfocus X-ray computed tomography

G. Kerckhofs[1, 2,+,*], M. Durand[1,2,3,4,+], R. Vangoitsenhoven[5], C. Marin[1, 2], B. Van der Schueren[5], G. Carmeliet[5], F. Luyten[1, 2], L. Geris[2,6,7], K. Vandamme[2,8]

Supplementary data

Mechanical testing

After harvesting, the femurs were kept at -80°C while being wetted in PBS-soaked cotton tissue (n = 4 per group). Prior to testing, the samples were thawed, while being kept humid. All the samples were subjected to a 3-point bending test using a custom-made 3-point bending set-up that was mounted on a BOSE Test Bench (LM1, EnduraTEC Systems Group, Bose Corp., Minnetonka, MN, USA). The femurs were placed on their posterior surface on the rigid supporting beams with rounded edges (1 mm diameter) to avoid shear loading and cutting. The length of the spam was 6 mm for all samples. The femurs were centred on the supporting beams and the pressing force was applied directly to their anterior side. Before the actual testing, a stabilizing pre-load of 1N and 3N was applied to the midshaft of the femurs. The bending load, a rate of 0.1mm/s, was applied to the femurs until failure.

From the load-displacement curves, the bending stiffness of the samples was determined (Suppl.Fig. S1). Normal ageing resulted in a significantly increased stiffness (36% – p=0.0001), while obesity-driven type 2 diabetes decreased the stiffness significantly (-17% – p=0.029).

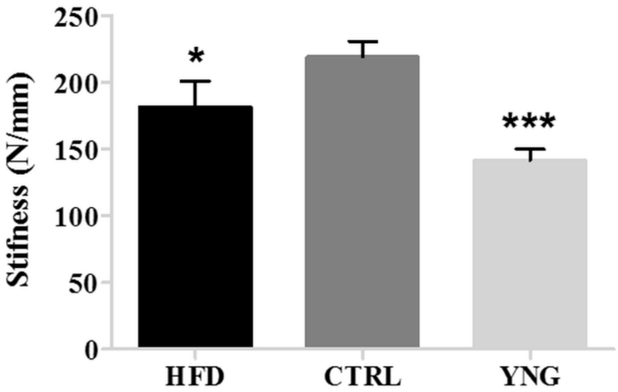

Supplementary figure S1: Bending stiffness, derived from a 3-point bending test, of femurs of the HFD, CTRL and YNG groups. n=4/group.

Sensitivity of the despeckling step

To evaluate the sensitivity of the despeckling step on the cortical lacunar porosity, lacunar diameter and number of lacunae, and to determine the most optimal despeckling value for our study, we ranged the white speckle volume from 220 µm³ up to 340 µm³ in steps of 30 µm³, and also included more extreme values (150 µm³, 450µm³ and 550 µm³). When comparing the cortical lacunar porosity, lacunar diameter and number of lacunae between the CTRL and HFD group and between the CTRL and YNG group, for none of the despeckling volumes significant differences were found, even not for the extreme values (Suppl.Fig. S2). Also, the same trends were noticed for the despeckling volumes from 150 µm³ up to 340 µm³, namely a decreasing trend in lacunar porosity and density due to ageing and HFD, and on average a lower lacunar diameter for the CTRL group compared to both the YNG and the HFD group, and this was confirmed by other studies. For the larger despeckling volume (450 µm³ and 550 µm³), the trends altered, suggesting that changing the despeckling volume within the range of 150 µm³ up to 340 µm³ did not have an impact on the final outcome.

Furthermore, the derivative of the average cortical lacunar porosity, lacunar diameter and number of lacunae for the three groups in function of the despeckling volume was determined.

For the first two parameters a maximum was found around 280-330 µm³, indicating that between these values, the influence of the despeckling value was the least. For the latter, however, no maximum was found. Based on these results, we selected a threshold of 280 µm³ as lower limit despeckling volume.

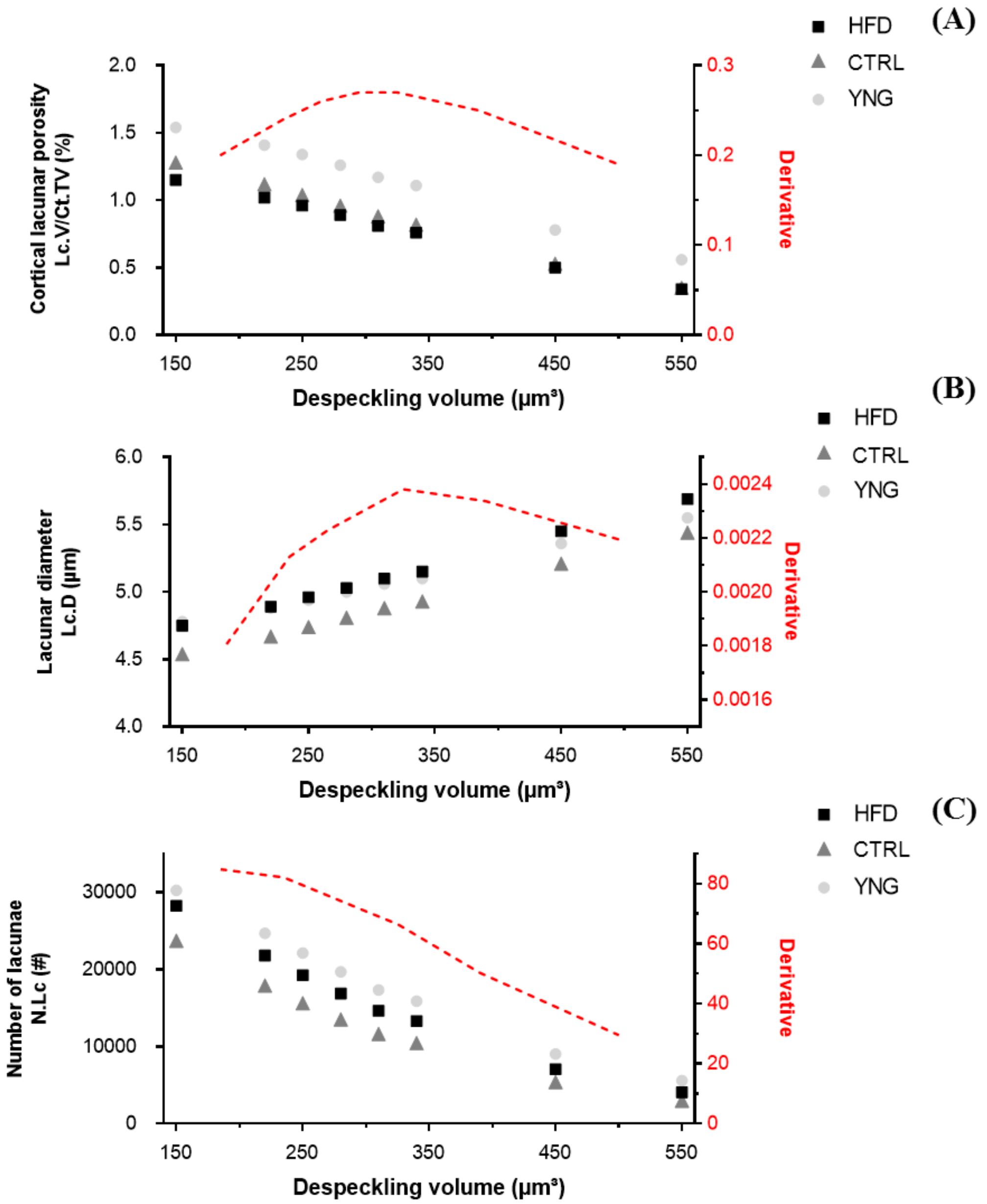

Supplementary figure S2: HR-microCT-based analysis of the (A) cortex lacunar porosity, (B) lacunar diameter and (C) number of lacunae density in function of the despeckling

volume for the HFD, CTRL and YNG groups, along with the derivative of this function averaged over the three animal groups. n=7-8/group.
